# Effects of Non-Covalent Functionalized Graphene Oxide with Hyperbranched Polyesters on Mechanical Properties and Mechanism of Epoxy Composites

**DOI:** 10.3390/ma12193103

**Published:** 2019-09-23

**Authors:** Jin Tian, Ting Xu, Yefa Tan, Zhongwei Zhang, Binghui Tang, Zhidan Sun

**Affiliations:** 1 Institute of Field Engineering, Army Engineering University of PLA, Nanjing 210007, China; tianjin156@163.com (J.T.); xuting156@163.com (T.X.); tangbinghui156@163.com (B.T.); sunzhidan156@163.com (Z.S.); 2 National Defense Engineering Institute, Army Engineering University of PLA, Nanjing 210007, China; zhangzhongwei156@163.com

**Keywords:** epoxy composites, graphene oxide, hyperbranched polyesters, mechanical properties, non-covalent functionalization

## Abstract

In order to improve the interfacial properties of graphene oxide (GO) and epoxy resin (EP), hyperbranched polyesters with terminal carboxyl (HBP) non-covalently functionalized graphene oxide (HBP-GO) was achieved by strong π-π coupling between hyperbranched polyesters and GO nanosheets. The effects of non-covalent functionalization of GO on the dispersibility, wettability and interfacial properties were analyzed. The mechanical properties and enhancement mechanism of HBP-GO/EP composites were investigated. The results show that the hyperbranched polyesters is embedded in the GO layer due to its highly branched structure, which forms the steric hindrance effect between the GO nanosheets, effectively prevents the agglomeration of GO nanosheets, and significantly improved the dispersibility of GO. Simultaneously, the contact angle of HBP-GO with EP is reduced, the surface energy, interfacial energy and adhesion work are increased, then the wetting property of HBP-GO is significantly improved. The main toughening mechanism of HBP-GO is microcrack deflection induced by HBP-GO and plastic deformation of the EP matrix. In the microcrack propagation zones, HBP-GO may produce the pinning effect near the microcrack tips and change their stress state, resulting in microcrack deflection and bifurcation. So, the microcrack propagation path is more tortuous, which will consume much more fracture energy. Therefore, the mechanical properties of the HBP-GO/EP composites are greatly improved.

## 1. Introduction

Epoxy resin has been applied extensively in structural adhesives, electronic instruments, construction, coatings and other fields due to its high mechanical properties, good bonding properties, low cure shrinkage and good process performance. In particular, epoxy resin has become an irreplaceable and important material as a composite matrix [1,2,3]. However, after curing, the epoxy resin forms a thermosetting polymer oligomer having a crosslinked molecular network, which has high brittleness and poor mechanical properties. It is difficult to adapt to the needs of modern industry [4,5,6,7]. Therefore, improving the mechanical properties of epoxy resins has become the research focus of this field. Recently, graphene oxide and graphene have become new materials of high concern in the scientific community due to their unique two-dimensional structure and excellent mechanical properties. In recent years, numerous studies have shown that the use of graphene oxide (GO), graphene or their derivatives as a polymer matrix composites reinforcing filler is remarkable in improving the mechanical properties of the polymer matrix [8,9,10,11]. However, because of the strong π-π coupling between the graphene nanosheets, irreversible agglomeration is highly prone to occur. Moreover, the wettability between the GO and the resin matrix is poor, and the interface bonding strength is weak, which is disadvantageous for bearing the load transmitted from the substrate [12,13,14].

The functionalized treatment of graphene oxide (GO) opens up a wide range of application for resin-based composites [15,16,17]. The covalent functionalization of the surface is generally effective in controlling the chemical properties of GO to improve stress transfer from the polymer to the GO nanosheets. For examples, Lin et al. [18] prepared covalently bonded GO (PE-g-GO) using PE grafted with γ-aminopropyltriethoxysilane and maleic anhydride. The results show that PE-g-GO is uniformly dispersed in the PE matrix, and the Young’s modulus, tensile strength and yield strength of the composites are significantly improved. Wan et al. [19] prepared covalently functionalized GO (silane-f-GO) via a silane with an epoxy group. The results show that the silane with terminal epoxy groups effectively improves the compatibility between the GO and the EP matrix, as well as the glass transition temperature, bending properties, tensile properties and toughness of the epoxy composites is significantly improved compared to the pure EP. Although some methods of covalent functionalization of graphene surfaces have proven effective, they create additional defects on the graphene surface that destroy the inherent structure of graphene. Furthermore, complex synthesis methods and chemical treatments are severely limited in engineering applications during the synthesis of derivatives. Thence, in order to make maximum use of the inherent structure and mechanical properties of graphene oxide or graphene, non-covalent functionalization is a more effective method [20,21,22]. Hyperbranched polymers (HBP) have a unique highly branched three-dimensional molecular structure. Compared to linear polymers, hyperbranched polymers with three-dimensional structure have lower melt, solution viscosity, better solubility, and higher functional groups density, which has great potential for application in functionalized GO [23]. Therefore, HBP have great potential for application in non-covalently functionalized GO. So far, there have been few reports on the dispersibility and mechanical properties of HBP non-covalently functionalized GO reinforced EP matrix composites.

In this study, hyperbranched polyester (HBP) non-covalently functionalized GO (HBP-GO) was prepared by using a relatively simple processing method, and the HBP-GO reinforced EP matrix composites were produced by solution casting. The infiltration property of HBP-GO was discussed by the test of contact angle, and the interface properties were analyzed by quantitative calculation of the interface energy and the adhesion work. The fracture morphologies of HBP-GO reinforced epoxy composites were observed, and the enhancement mechanism of HBP-GO was expounded, which provided a reliable basis for the application of GO-reinforced resin-based composites in engineering.

## 2. Materials and Methods 

### 2.1. Materials

Graphene oxide (GO, 95%) used in this experiment was supplied by Nanjing JCNano Technology Co., Ltd., Nanjing, China. Hyperbranched polyesters (HBP-COOH) is supplied by WH Hyperbranched Resin Technology Co., Ltd., Wuhan, China. The epoxy resin (E51) was supplied by Wuxi Resin Factory Co., Ltd., Wuxi, China. The QS-1622 curing agent is provided by Beijing Qingda Qi Shi New Material Technology Co., Ltd., Beijing, China. (QS-1622 and E51 compound ratio is 1:2). *N*,*N*-Dimethylformamide (DMF), tetrahydrofuran (THF) and absolute ethanol were obtained from Shanghai Aladdin Biochemical Technology Co., Ltd., Shanghai, China.

### 2.2. Preparation of Non-Covalent Functionalized GO

First, 100 mg of HBP-COOH was dissolved in 20 mL of DMF. Then it was mixed with the DMF dispersion of GO and stirred with the ultrasonic dispersion at 24 °C for 1 h. Then the GO dispersion was allowed to stand for 1 h. Next, filtration was carried out using a polycarbonate (PC) with a diameter of 10 μm under the condition of low pressure and the functionalized GO was washed with excess absolute ethanol. The filtration and washing process were repeated 3 times, and the finally obtained product was dried in a vacuum oven until the weight is stable to obtain a hyperbranched polyester functionalized GO (HBP-GO).

### 2.3. Preparation of HBP-GO Reinforced Epoxy Composites

First, HBP-GO was dispersed in a THF solution and sonicated for 2 h. After sonication, the dispersion solution of HBP-GO/THF was prepared. Then, the dispersion solution of HBP-GO/THF was added into the EP. The mixture was sonicated at 60 °C for 2 h while the mixture was stirred at 2000 rpm using the high speed mechanical mixer. Finally, the mixture was degassed in vacuum for 24 h and then mixed with QS-1622 curing agent. After stirring for 10 min and evacuating at 70 °C for 1 h, the mixture was poured into the PTFE mold for curing. Moreover, in order to study the enhanced mechanism of functionalized graphene oxide reinforced EP (HBP-GO/EP), untreated graphene oxide enhanced EP (GO/EP) and pure EP samples were also prepared for comparison. The distribution is shown in Table 1.

### 2.4. Characterization

The test result of FITR spectra were obtained using the Tensor 27 manufactured by Bruker Manufacturing, Germany. The scanning resolution is 4 cm^−1^, the scanning time is 32 times, and the scanning range is 500–4000 cm^−1^. The TGA was tested by a PL thermal analyzer (SDTA851, Mettlertoledo, UK). The test conditions were a nitrogen atmosphere with a heating rate of 20 °C/min. Scanning electron microscopy (SEM) measurements were carried out with a Hitachi S-4800 scanning electron microscope, city, country. Before this step, the tensile section of the GO/EP composite material needs to be sprayed with gold to enhance its electrical conductivity. The state of dispersion of GO and HBP-GO in the EP matrix was obtained by JEM-2100, JEOL transmission electron microscopy, city, country. The ultra-thin sections of the GO/EP composites sample with a size of 1 mm × 1 mm and a thickness of 50–60 nm was prepared by a diamond knife. The contact angle of the GO film with EP was performed by the optical meter OCA20, Dataphysics. The GO film was obtained by filtering under reduced pressure from a GO/THF dispersion having a content of 1 mg/ml. The filter membrane was polycarbonate (PC) with a diameter of 10 μm.

The Shore hardness was obtained by the D-type tester. The fracture properties of the epoxy composite were tested in accordance with ASTM D5045-99 using a rectangular parallelepiped three-point bending specimen (SENB) having a sample size of 50 × 10 × 5 mm^3^. The pre-cracking method of the sample is as follows: Firstly, a groove of 2 mm deep is processed by a small hand saw, and then the pre-formed crack is introduced into the bottom of the groove of the rectangular parallelepiped sample by a sharp blade. The experimental temperature was room temperature and the experimental loading rate was 1 mm/min. furthermore, at least 5 samples should be tested for the same component. The formula of fracture toughness (*K_IC_*) and critical energy release rate (*G_IC_*) is as follows [24]:

(1) $$K_{IC}=G\frac{P_{Q}S}{4BW^{3/2}},$$

(2) $$G_{IC}=\frac{\left(1-\nu^{2}\right)}{E}K_{IC}^{2},$$

where, *G* is the shape factor of the microcracks, *B* is the sample thickness, *E* is the sample elastic modulus, *W* is the sample width and *S* is the span. Both the EP and its composites have a Poisson’s ratio (*v*) of 0.35 [25].

## 3. Results and Discussion

### 3.1. Characterization of Non-Covalent Functionalized Graphene Oxide

**Figure 1:**
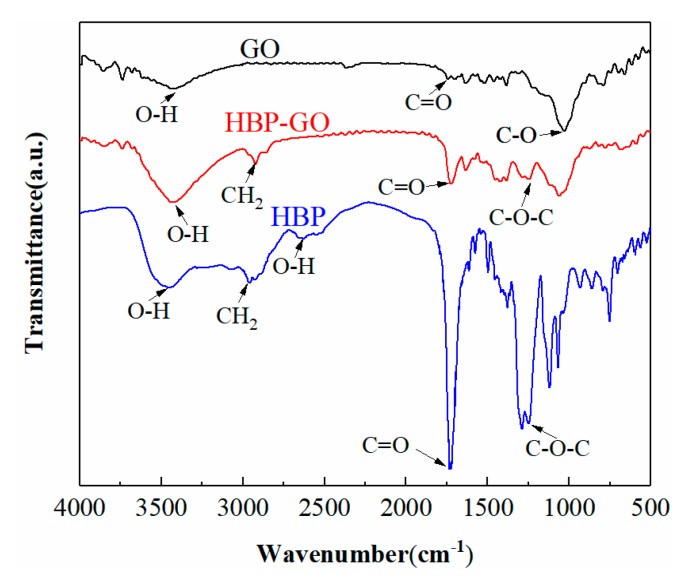
FT-IR spectra of GO, HBP and HBP-GO.

The FT-IR spectra of GO, HBP and HBP-GO are shown in Figure 1. For the GO nanosheets, there are some characteristic bands at 3354 cm^−1^ (O-H stretch of carboxylic acid and hydroxyl), 1715 cm^−1^ (C=O stretch) and at 1049 cm^−1^ (C-O-C stretch). The FTIR spectrum of HBP shows characteristic peaks of methylene stretch in the range of 2853–2955 cm^−1^. As well, there is the stretching peak of O-H in 2520–2640 cm^−1^ range and the stretching peak of C=O in carboxylic acid groups at 1639cm^−1^. In addition, the peak for C=C stretching of the benzene ring of HBP is observed in the range of 1495–1611 cm^−1^. As well, 1230 cm^−1^ is ascribed to the stretching characteristic peak of C-O-C. Compared with the peak of unmodified GO, the HBP-GO nanosheets showed obvious methylene characteristic peak at 2923 cm^−1^. Moreover, C-O-C stretching vibration peak appeared at 1230 cm^−1^, and the O-H stretch and C=O stretch were obviously enhanced. These new absorption peaks generated on the HBP-GO indicate that HBP has been adsorbed on GO, and the HBP-GO has been successfully prepared.

**Figure 2:**
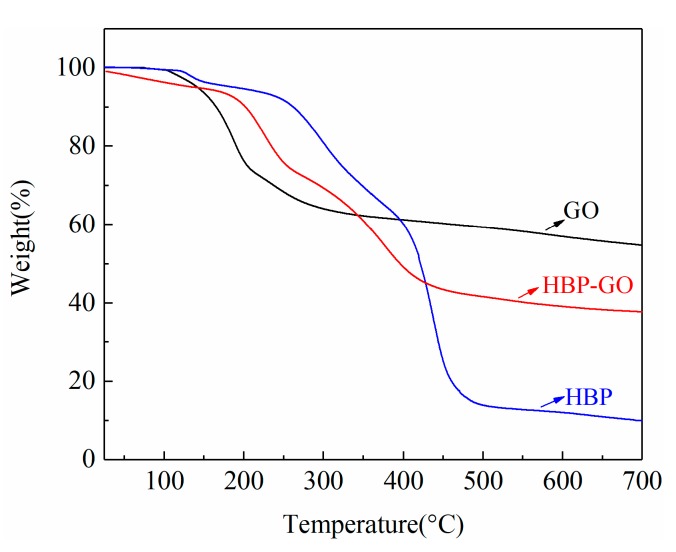
TGA results of GO, HBP and HBP-GO.

The TGA test results of GO, HBP and HBP-GO are shown in Figure 2. The mass of HBP adsorbed on the GO can be calculated by analyzing the TGA curve. As shown in Figure 2, the thermal weight loss of GO in the range of 160–240 °C is greatly increased due to the decomposition of a large number of unstable oxygen-containing functional groups on the GO nanosheets (OH and COOH). Meanwhile, a stable weight loss was observed at 300–700 °C, which can be attributed to the decomposition of the carbon structure of GO and the residual weight was about 54.75 wt%. According to the TGA curve of HBP-GO, the thermal stability of HBP-GO is better than that of GO. The initial decomposition temperature increases from 160 °C to 220 °C, and the sharp decomposition temperature occurs in the range of 230–380 °C. By comparing the TGA test results of HBP and HBP-GO, it can be found that both of them decompose violently near 380 °C and tend to be stable at 600 °C, which indicates that the weight loss occurring around 380 °C is mainly due to the decomposition of HBP adsorbed on the surface of GO and the residual weight of HBP-GO was about 37.72 wt%. Combined with the final residue weight of HBP, it can be calculated that the quantity of HBP adsorbed on GO is about 38.14 wt%.

### 3.2. HBP-GO Morphology and Their Dispersion in Epoxy Resin

**Figure 3:**
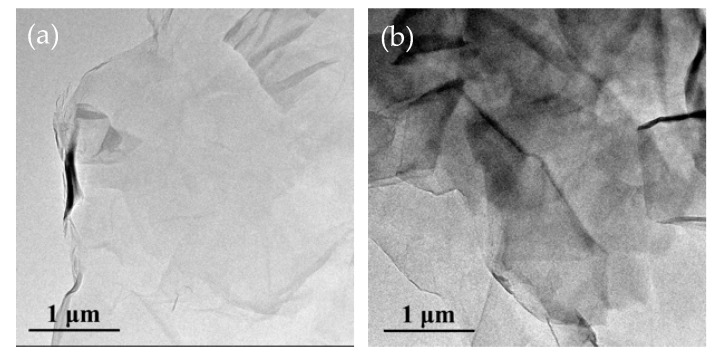
TEM images of GO and HBP-GO. (**a**) GO; (**b**) HBP-GO.

The morphologies of GO and HBP-GO were researched by TEM imaging, as shown in Figure 3. The surface of GO is smooth and has a highly transparent sheet-like morphology due to its structure with a small atomic thickness (Figure 3a). The size of GO is about 3–5 μm, and it exhibits a slight wrinkle and folded structure due to its abundant oxidizing groups. After functionalization, the HBP-GO surface became rough and there are a large number of black opaque regions distributed on the surface of HBP-GO (Figure 3b). These phenomena indicate that HBP successfully adheres to the GO surface after functionalization.

**Figure 4:**
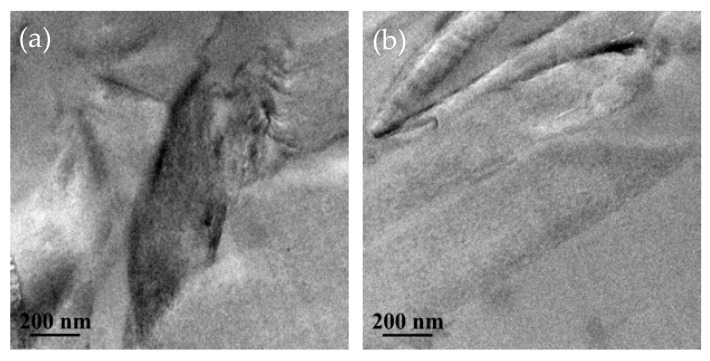
TEM images of GO/EP and HBP-GO/EP. (**a**) GO/EP; (**b**) HBP-GO/EP.

In order to further study the dispersion state of GO and HBP-GO in EP matrix, ultrathin sections of GO/EP and HBP-GO/EP composites were observed by TEM (Figure 4). Some dark areas with different sizes can be clearly observed in Figure 4a, indicating that the GO agglomerates in the EP matrix. After functionalization, HBP-GO was uniformly dispersed in the EP matrix, and no agglomerates were formed (Figure 4b), indicating that HBP-GO has good dispersion properties in the EP matrix, which can be attributed to the well compatibility of HBP-GO.

### 3.3. The wetting and Interface Properties between HBP-GO and EP

**Figure 5:**
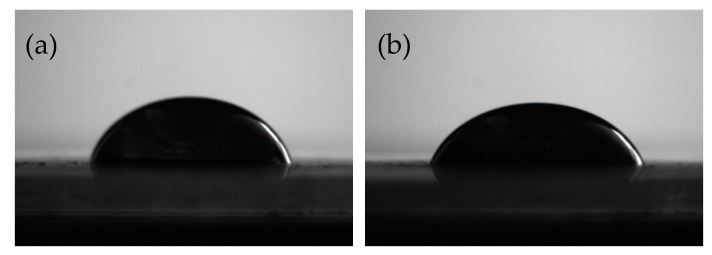
Contact angle of EP droplets on GO and HBP-GO. (**a**) GO (**b**) HBP-GO.

Contact angle could characterize the wetting properties of GO and EP matrix. By measuring the contact angle of GO and EP before and after functionalization, the wettability can be visually indicated. The measurement results are shown in Figure 5. It can be found that the contact angle of GO with EP is about 61.6°, and after HBP functionalization, the contact angle of HBP-GO with EP is reduced to 56.7°. This indicates that the infiltration property between the HBP-GO and EP is obviously improved, and it can effectively reduce the defects at the interface. Therefore, the interfacial adhesion strength between the HBP-GO and the EP matrix is improved, which will contribute to the improvement of the mechanical properties of the epoxy composites.

In order to analyze the interface properties of HBP-GO, the interface energy and adhesion work were analyzed and calculated. According to the Young-Dupre equation [26], the contact angle (*θ*), adhesion work, and surface energy can be obtained by the formula:

(3) $$\gamma_{LS}=\gamma_{S}-\gamma_{L}\cos\theta ,$$

(4) $$W_{a}=\gamma_{L}+\gamma_{S}-\gamma_{LS}=\gamma_{L}(1+\cos\theta ),$$

where, *γ_L_*, *γ_S_*, and *γ_LS_* represent the surface energy of the liquid, solid, and the interface of solid-liquid. *W_a_* represents the adhesion work of the interface of solid-liquid. Based on Owens, D.K.J and Wendt, R.C.J [27], the interface energy of solid-liquid can be calculated by the following formula:

(5) $$\gamma_{LS}=\gamma_{S}+\gamma_{L}-2\sqrt{\gamma_{S}^{p}\gamma_{L}^{p}}-2\sqrt{\gamma_{S}^{d}\gamma_{L}^{d}},$$

(6) $$\gamma_{S}=\gamma_{S}^{p}+\gamma_{S}^{d},$$

(7) $$\gamma_{L}=\gamma_{L}^{p}+\gamma_{L}^{d},$$

where, *γ_S_^p^* and *γ_S_^d^* are the polar and non-polar components of solid surface energy, and *γ_L_^p^* and *γ_L_^d^* are the polar and non-polar components of liquid surface energy.

From Equations (3) and (5), Equation (8) is available:

(8) $$\gamma_{L}(\cos\theta +1)=2\sqrt{\gamma_{S}^{p}\gamma_{L}^{p}}+2\sqrt{\gamma_{L}^{d}\gamma_{L}^{d}},$$

According to the Equation (8), two kinds of solvents known as *γ_L_*, *γ_L_^p^* and *γ_L_^d^* are selected to calculate the surface energy of GO. The solvent used in the experiment was glycerol and water with a large difference in surface energy components. The *γ_L_^p^* and *γ_L_^d^* of glycerol were 21.7 mJ/m^2^ and 41.9 mJ/m^2^, respectively, and the *γ_L_^p^* and *γ_L_^d^* of water were 52.1 mJ/m^2^ and 20.8 mJ/m^2^, respectively. Moreover, according to previous studies, the surface energy of EP is 41.5 mJ/m^2^ [28]. The calculation results obtained by the Equations (3), (4) and (8) are listed in Table 2 and Table 3.

The surface energy calculation results of GO and HBP-GO are listed in Table 2. The results showed that the surface energy of original GO was 48.41 mJ/m^2^. After functionalization with HBP, the surface energy of HBP-GO was significantly improved, which was 23.2% higher than that of original GO. By comparing the surface energy components of GO and HBP-GO, we can know that the increase of surface energy for HBP-GO is mainly because the improvement of non-polar components. The non-polar component of surface energy is caused by the interaction between the electron dipole and the induced dipole in the neighboring atom or molecule [29]. Due to the smooth surface of the original GO (Figure 3a), the dipole interaction is weak, resulting in a low non-polar component of surface energy (41.86 mJ/m^2^). After functionalization with HBP, the non-polar component of HBP-GO surface energy increases to 49.98 mJ/m^2^ due to the strong π-π interaction, which also gives HBP-GO a larger surface energy (59.65 mJ/m^2^). Meanwhile, the non-polar component of the surface energy reflects the non-polar part of the van der Waals force, which can characterize the morphology of the solid surface. Therefore, the increase of the non-polar component of HBP-GO surface energy indicates that HBP functionalized GO increases the GO surface roughness, which is consistent with the above-mentioned research results (Figure 3b).

Compared the interface energy and the adhesion work of the GO before and after functionalization (Table 3), it can be seen that, after functionalization, the interface energy and the adhesion work between HBP-GO and EP are improved and reach 37.14 mJ/m^2^ and 63.51 mJ/m^2^ respectively, which are increased by 28.6% and 5.7% than those of the GO (28.91 mJ/m^2^ and 60.50 mJ/m^2^).

### 3.4. Mechanical Properties

#### 3.4.1. Microhardness

**Figure 6:**
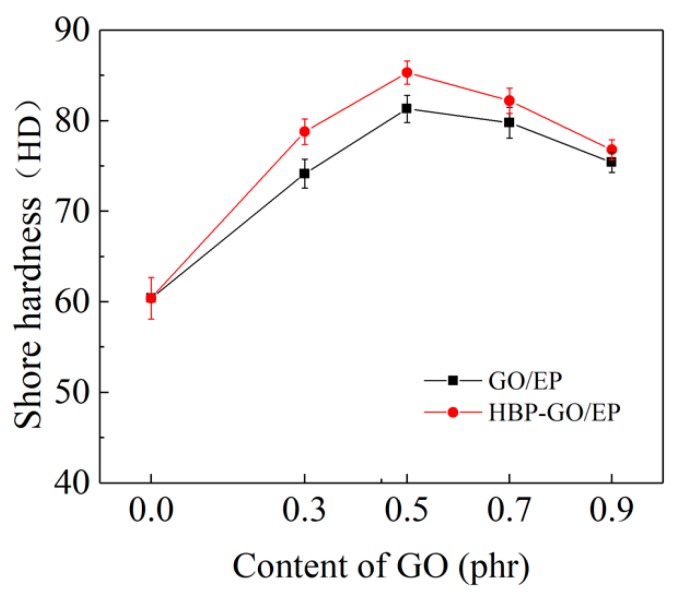
Shore hardness of GO/EP and HBP-GO/EP.

Figure 6 shows the results of microhardness testing of EP, GO/EP and HBP-GO/EP, respectively. It can be seen that the microhardness of GO and HBP-GO reinforced epoxy composites is improved compared with pure EP. The microhardness of the 0.5 phr HBP-GO/EP composites reaches the maximum value of 84.5 HD, which was 40.83% higher than that of pure EP and was 5.12% higher than the 0.5 phr GO/EP composites. This is because the GO nanosheets possess high strength (75 GPa) and the their dispersibility in the epoxy composite may affect their bearing force state. When they are pressed by external load, the uniformly dispersed HBP-GO nanosheets in the EP matrix can effectively bear part of the load, which enhances the ability of the HBP-GO/EP composites to resist plastic deformation or damage, which effectively improve the microhardness of the EP composites.

#### 3.4.2. Tensile Properties

**Figure 7:**
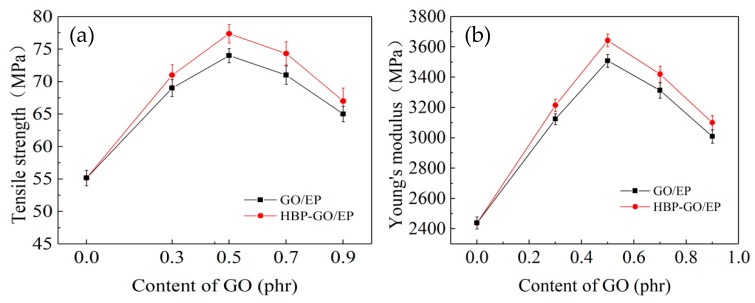
Tensile properties of the epoxy composites. (**a**) Tensile strength and (**b**) young’s modulus.

The test results of tensile properties of different content of GO nanosheets reinforced epoxy composites are shown in Figure 7. Obviously, as the HBP-GO content increases from 0.3 phr to 0.9 phr, the tensile strength and Young’s modulus of the HBP-GO/EP composites exhibit a tendency to increase first and then decrease. When the HBP-GO content is 0.5 phr, the tensile strength and Young’s modulus of the epoxy composite (0.5 phr HBP-GO/EP) reach the maximum values (77.8 MPa and 3658.34 MPa), which are increased by 41.4% and 49.9% than those of the EP. Also, compared with the unmodified GO reinforced epoxy composites (GO/EP), they are increased by about 6.5% and 5.7%, respectively. This indicates that the non-covalent functionalization method adopted in this paper significantly improves the enhancement effect of GO. However, as the content of HBP-GO increases (0.9 phr), the enhancement effect will decrease because a part of HBP-GO produces agglomeration. According to the above results, the enhancement effect of HBP-GO may be derived from the following two reasons. Firstly, the dispersibility of HBP-GO is effectively improved after non-covalent functionalization. HBP-GO can withstand loads more effectively in the EP matrix compared with the untreated GO shown in Figure 4a. Secondly, HBP-GO/EP achieved an enhanced effect due to the improvement of interfacial energy and adhesion between HBP-GO and EP.

In order to predict the elastic modulus of the epoxy composite, we assume that the GO nanosheet is a rectangular cross-section fiber with width (*W*), length (*L*), and thickness (*t*). The Halpin-Tsai equation of randomly oriented fibers is applied as follows [30,31,32,33,34]:

(9) $$\alpha =\frac{E_{C}}{E_{M}}=\frac{3}{8}\left(\frac{1+\zeta \eta_{L}V_{GO}}{1-\eta_{L}V_{GO}}\right)+\frac{5}{8}\left(\frac{1+2\eta_{W}V_{GO}}{1-\eta_{W}V_{GO}}\right),$$

(10) $$\eta_{L}=\frac{\beta -1}{\beta +\zeta },$$

(11) $$\eta_{W}=\frac{\beta -1}{\beta +2},$$

where, *E_C_* and *E_M_* represent the modulus of composites and the pure EP; α represents the ratio of *E_C_* to *E_M_*. *V_GO_* is the volume fraction of GO. The GO volume fraction is converted to a mass fraction according to the standard density of GO (F = 2.25 g/cm^3^) and the matrix density (F = 1.12 g/cm^3^) listed by the manufacturer. *β* and *ζ* are constants:

(12) $$\beta =\frac{E_{GO}}{E_{M}},$$

(13) $$\zeta =\frac{W+L}{t},$$

**Figure 8:**
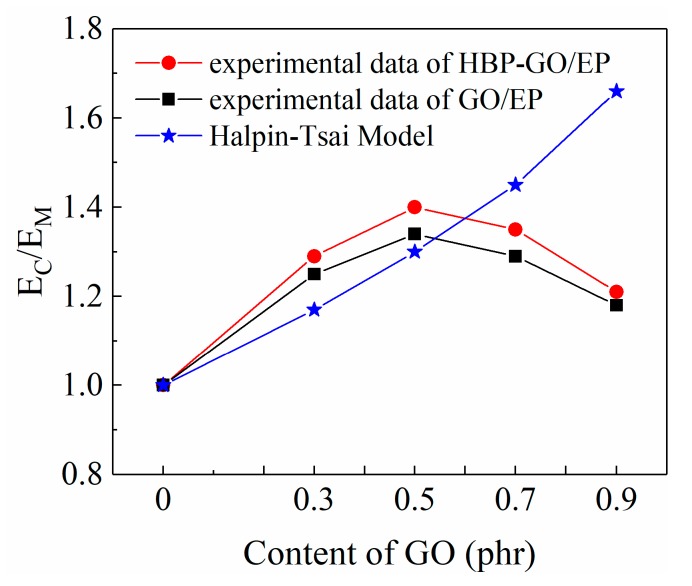
The predicted and experimental results of elastic modulus of the composites.

Substituting from Equations (10)–(13) into Equation (9), α can be calculated by the following formula:

(14) $$\alpha =\frac{E_{C}}{E_{M}}=\frac{3}{8}\left(\frac{1+\left(\left(W+L\right)/t\right)\frac{\left(E_{GO}/E_{M}\right)-1}{\left(E_{GO}/E_{M}\right)+\left(\left(W+L\right)/t\right)}V_{GO}}{1-\frac{\left(E_{GO}/E_{M}\right)-1}{\left(E_{GO}/E_{M}\right)+\left(\left(W+L\right)/t\right)}V_{GO}}\right)+\frac{5}{8}\left(\frac{1+2\frac{\left(E_{GO}/E_{M}\right)-1}{\left(E_{GO}/E_{M}\right)+2}V_{GO}}{1-\frac{\left(E_{GO}/E_{M}\right)-1}{\left(E_{GO}/E_{M}\right)+2}V_{GO}}\right),$$

where *E_GO_* = 1 TPa, *E_M_* = 2.99 GPa. Since GO nanosheets have no defined length and width, it is necessary to introduce an approximate size estimate based on TEM characterization, assuming that the average *W*, *L*, and *t* are equal to 3 μm, 5 μm, and 4 nm. The theoretical calculations and experimental modulus are compared in Figure 8.

There is a reasonable agreement between the predicted and experimental values until 0.5 phr of GO for the composites. When it is bigger than 0.5 phr, the experimental modulus begins to fall below the prediction. As is shown in Figure 8, when the content of GO reaches 0.9 phr, there is a large deviation between the predicted and experimental modulus, and the test modulus is much lower than the predicted modulus. Moreover, the experimental modulus of HBP-GO/EP composites is higher than that of GO/EP. This trend could be explained by two circumstances. Firstly, the dispersibility of HBP-GO in EP began to decrease when the content of HBP-GO exceeded 0.5 phr. Secondly, after functionalization with HBP, the dispersibility and interfacial properties of HBP-GO in the epoxy group are significantly improved. In summary, under the condition of uniform dispersion of HBP-GO, Halpine-Tsai can be used to predict the lower limit of the experimental modulus of the epoxy composites.

#### 3.4.3. Fracture Toughness

**Figure 9:**
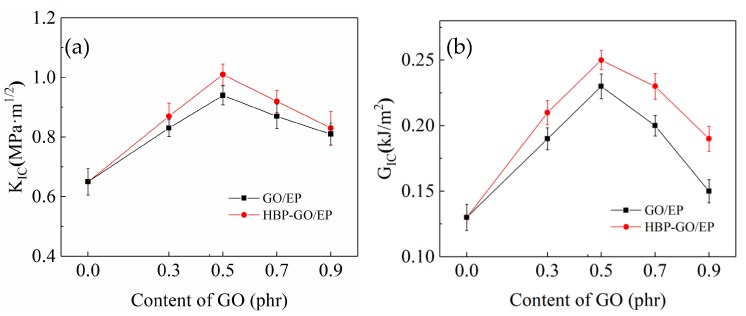
Fracture properties of GO/EP and HBP-GO/EP composites. (**a**) *K_IC_*; (**b**) *G_IC_*.

Figure 9 shows the test results of the *K_IC_* and *G_IC_* of GO and HBP-GO reinforced epoxy resin composites. It can be found that the *K_IC_* and *G_IC_* of the pure EP are 0.75 MPa·m^1/2^ and 0.19 KJ/m^2^, respectively. The *K_IC_* and *G_IC_* of GO/EP and HBP-GO/EP are higher than those of pure EP and the HBP-GO has a better enhancement effect. When the HBP-GO content is 0.5 phr, the *K_IC_* and *G_IC_* of the HBP-GO/EP composites reach 1.01 MPa·m^1/2^ and 0.26 kJ/m^2^, which are increased by 35.7% and 36.8% than the pure EP and increased by 7.45% and 13.04% than those of 0.5 phr GO/EP (0.94 Mpa·m^1/2^ and 0.23 kJ/m^2^). The reasons are as follows: Firstly, This is because HBP-GO is dispersed in the EP matrix uniformly and the interfacial properties with the resin matrix is pretty good (Figure 4b). Secondly, the HBP on the surface of the GO can coordinately deform with the epoxy system during the curing process. It absorbs more energy and provides sufficient guarantee for the toughening effect of GO. Therefore, HBP-GO has an effective toughening effect on epoxy resin, and HBP-GO/EP composite has higher fracture toughness.

Based on the equation of Griffith (15), the size of critical microcracks to different samples can be calculated [35]:

(15) $$C=\frac{2K_{IC}^{2}}{\pi \sigma^{2}},$$

where, *C* and *σ* represent the critical microcrack length and the tensile strength of the samples. 

**Figure 10:**
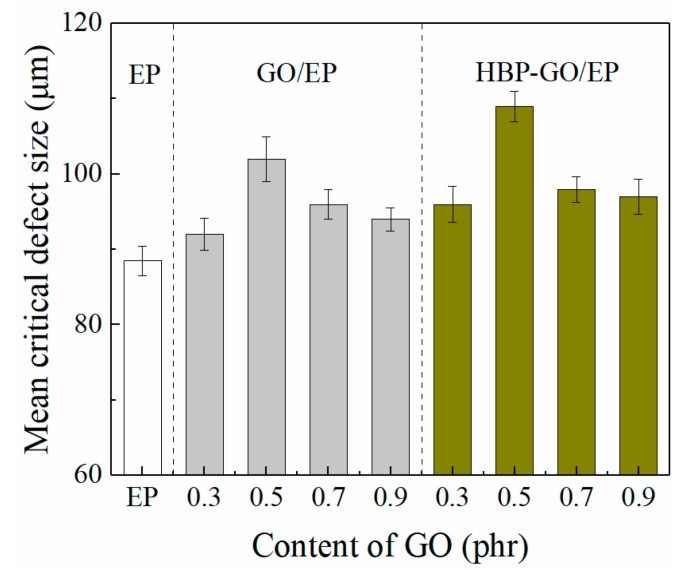
Mean critical crack length of the composites.

Figure 10 shows the average critical microcrack length of the composites. It can be found that for the pure EP, the critical microcrack length is approximately 88.5 μm, which is the smallest in all the tested samples. Both GO and HBP-GO can increase the critical crack length of EP, in which HBP-GO has a better enhancement effect. When the HBP-GO content is 0.5 phr, the critical crack length of the HBP-GO/EP composite reaches a maximum of 109 μm, which is 120% bigger than that of the pure EP and 6.7% bigger than the GO/EP (102 μm). This is because HBP-GO helps to release the internal stress of the material, improving the interface between the HBP-GO and the matrix, thereby increasing the critical crack length.

#### 3.4.4. Analysis of the Fracture Surface of HBP-GO/EP

**Figure 11:**
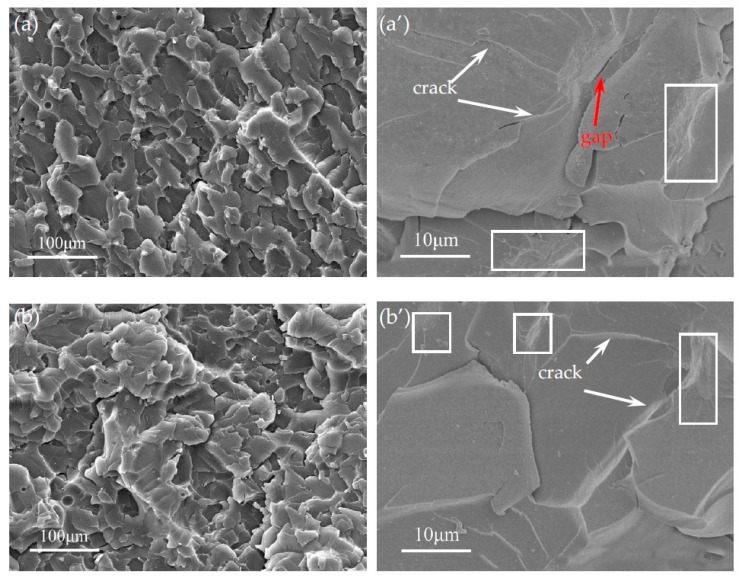
SEM images of the fracture surfaces. (**a**) GO/EP, (**b**) HBP-GO/EP, (**a’**), (**b’**) are the high magnification of GO/EP and HBP-GO/EP.

The morphologies of the fracture surfaces of GO/EP and HBP-GO/EP are shown in Figure 11. It can be seen from the cross-sectional morphology of GO/EP (Figure 11a) that there is a large amount of dimple-like morphology on the cross-section, which is a typical fracture morphology of graphene and graphene derivative reinforced composites reported in previous literatures. Also, some micron-scale agglomerates (Figure 11a’) can be seen on the cross section, indicating poor dispersion. [36,37]. In addition, it can be found from the high magnification of these dimples (Figure 11a’) that the cracks are deflected or bifurcated when they encounter GO. Therefore, the toughening mechanism of GO is mainly microcrack deflection and bifurcation mechanism. However, a larger size of GO agglomerates appeared on the fracture surface of the GO/EP composite (Figure 11a’ rectangular frame) and a significant gap was observed between the GO and the epoxy resin matrix (red arrow in Figure 11a’), indicating that the interfacial adhesion between the GO and the epoxy resin matrix is weak and the dispersion property is poor, which severely limits the enhancement effect of GO.

The tensile fracture surface of the HBP-GO/EP composite is shown in Figure 11b. It can be seen that compared with the GO/EP composites, more dimple-like pits appear on the cross section of the HBP-GO/EP, and the fracture surface is rougher. The high magnification SEM image of Figure 11b’ shows that HBP-GO achieved good dispersion in the resin matrix and no significant agglomeration was observed. Moreover, the microcrack propagation path is more tortuous, and a large number of microcrack deflections and bifurcations occur. This is because HBP-GO may produce the pinning effect near the microcrack tips and change their stress state, resulting in microcrack deflection and bifurcation. So the microcrack propagation path is more tortuous, which can consume much more fracture energy. Therefore, the mechanical properties of the HBP-GO/EP composites are greatly improved. In addition, no gap is observed between the HBP-GO sheets and the EP matrix on the fracture surface. This indicates that the functionalized treatment improves the interfacial adhesion between the HBP-GO sheets and the EP matrix and promotes the HBP-GO to efficiently induce the deflection and bifurcation of the microcracks, showing the most excellent enhancement and toughening effect.

## 4. Conclusions

(1) HBP-GO was successfully prepared using hyperbranched polyesters (HBP) by a non-covalent functionalization method. the hyperbranched polyesters is embedded in the GO layer due to its highly branched structure, which forms the steric hindrance effect between the GO nanosheets, effectively prevents the agglomeration of GO nanosheets, and significantly improved the dispersibility of GO. Simultaneously, the contact angle of HBP-GO with EP is reduced, the surface energy, interfacial energy and adhesion work are increased, then the wetting property of HBP-GO is significantly improved. The tensile strength, elastic modulus, *K_IC_* and *G_IC_* of 0.5 phr HBP-GO reinforced epoxy resin composites reached 77.8 MPa, 3658.34 MPa, 1.01 MPa·m^1/2^ and 0.26 kJ/m^2^, respectively. Compared with pure epoxy resin, they were increased by 41.4%, 49.9%, 35.7% and 36.8%, respectively.

(2) The fracture surface analysis showed that HBP-GO was uniformly dispersed in the EP matrix and the fracture surface was rough. The toughening mechanism of HBP-GO is the deflection and bifurcation of crack induced by GO and plastic deformation of the matrix. In the microcrack propagation zones, HBP-GO may produce the pinning effect near the microcrack tips and change their stress state, resulting in microcrack deflection and bifurcation. So, the microcrack propagation path is more tortuous, which can consume much more fracture energy. Therefore, the mechanical properties of the HBP-GO/EP composites are greatly improved.

## Figures and Tables

**Table 1:** Formulation of the epoxy composites.

Series	Matrix (phr)	GO (phr)	HBP-GO (phr)
EP	100	-	-
GO/EP	100	0.3	-
	100	0.5	-
	100	0.7	-
	100	0.9	-
HBP-GO/EP	100	-	0.3
	100	-	0.5
	100	-	0.7
	100	-	0.9

**Table 2:** Contact angles and surface energy of various droplets on GO and HBP-GO.

	Contact Angles (°)		Surface Energy (mJ/m^2^)		
	Glycerol	Water	γ_S_^p^	γ_S_^d^	γ_S_
GO	46.2	70.5	6.55	41.86	48.41
HBP-GO	26.8	59.6	9.67	49.98	59.65

**Table 3:** Interface performance of GO/EP and HBP-GO/EP.

	GO	HBP-GO
Contact angle (°)	61.6 ± 1.0	56.7 ± 0.7
Interfacial energy (mJ/m^2^)	28.91	37.14
Adhesion work (mJ/m^2^)	60.50	63.51
